# Multi-Cue Event Information Fusion for Pedestrian Detection With Neuromorphic Vision Sensors

**DOI:** 10.3389/fnbot.2019.00010

**Published:** 2019-04-02

**Authors:** Guang Chen, Hu Cao, Canbo Ye, Zhenyan Zhang, Xingbo Liu, Xuhui Mo, Zhongnan Qu, Jörg Conradt, Florian Röhrbein, Alois Knoll

**Affiliations:** ^1^College of Automotive Engineering, Tongji University, Shanghai, China; ^2^Robotics, Artificial Intelligence and Real-time Systems, Technische Universität München, München, Germany; ^3^State Key Laboratory of Advanced Design and Manufacturing for Vehicle Body, Hunan University, Changsha, China; ^4^Computer Engineering and Networks Lab, ETH Zurich, Zurich, Switzerland; ^5^Department of Computational Science and Technology, KTH Royal Institute of Technology, Stockholm, Sweden

**Keywords:** neuromorphic vision sensor, event-stream encoding, object detection, convolutional neural network, multi-Cue event information fusion

## Abstract

Neuromorphic vision sensors are bio-inspired cameras that naturally capture the dynamics of a scene with ultra-low latency, filtering out redundant information with low power consumption. Few works are addressing the object detection with this sensor. In this work, we propose to develop pedestrian detectors that unlock the potential of the event data by leveraging multi-cue information and different fusion strategies. To make the best out of the event data, we introduce three different event-stream encoding methods based on Frequency, Surface of Active Event (SAE) and Leaky Integrate-and-Fire (LIF). We further integrate them into the state-of-the-art neural network architectures with two fusion approaches: the channel-level fusion of the raw feature space and decision-level fusion with the probability assignments. We present a qualitative and quantitative explanation why different encoding methods are chosen to evaluate the pedestrian detection and which method performs the best. We demonstrate the advantages of the decision-level fusion via leveraging multi-cue event information and show that our approach performs well on a self-annotated event-based pedestrian dataset with 8,736 event frames. This work paves the way of more fascinating perception applications with neuromorphic vision sensors.

## 1. Introduction

The rapid development of Artificial Intelligence technology drives the research on autonomous driving technology to be a hot spot. A reliable object detector should be a must in developed autonomous driving systems because more than half of road traffic accidents are related to pedestrians, motorcyclists, and bicyclists. It is vital for autonomous and semi-autonomous vehicles to detect them accurately within a short period. There are many approaches to pedestrian detection for the past decades. Conventional imaging sensors and time-of-flight sensors are the most commonly used sensing device for the pedestrian detection. The output data of these sensors could be effectively processed by several existing algorithms (Dollár et al., 2014; Ren et al., 2015; Liu et al., 2016b; Lin et al., 2017). The faster the algorithms can be, the more response time drivers or autonomous driving vehicles can hold to avoid possible collisions. However, most of the existing pedestrian detection systems based on these sensors fail to perform well in a real environment due to high latency, heavy calculation demanding, and relatively low accuracy. Moreover, time-of-flight sensors such as LiDARs are often too expensive to be utilized in such scenarios, while conventional imaging sensors often suffer from data redundancy, high latency, and ineffective under poor lighting conditions.

With the advent of neuromorphic vision sensors, we get another type of vision sensor for object detection (Liu et al., 2016a; Ramesh et al., 2017; Cannici et al., 2018). Compared to traditional cameras, neuromorphic vision sensor doesn't have frame rate concept and it supplies events based on pixel intensity changes. Dynamic Vision Sensor (DVS) is a type of neuromorphic vision sensors and outputs asynchronous events recording the illumination changes (Lichtsteiner et al., 2008; Posch et al., 2011; Berner et al., 2013). To be specific, a single event is recorded as a tuple (t, x, y, p), where x, y are the pixel coordinates of the event in 2D space, *t* is the time-stamp of the event, and *p* is the polarity of the event indicating the brightness change. Since the data of neuromorphic vision sensors is a spatiotemporal event stream, it significantly reduces data redundancy compared to conventional vision sensors, and achieves low latency, wide dynamic range and low power consumption. However, object detection using neuromorphic vision sensors is at the stage that is relatively initial due to the lack of annotated event-based datasets and the naive algorithms for neuromorphic vision sensors data. Lagorce et al. (2017) proposed a hierarchical recognition architecture which uses the spatiotemporal information from neuromorphic vision sensor to build features. Liu et al. (2016a) combined Active Pixel Sensor (APS) images which are grayscale and event frames to build a detector whose accuracy reaches 90%. However, since the frame rates of APS images are relatively low, they sacrifice the low latency characteristic of DVS.

Since CNNs perform well in object detection based on traditional vision sensors, we are trying to detect objects using this method with neuromorphic vision sensors. Chen (2018) use APS images on a Recurrent Rolling convolutional neural network to produce pseudo-labels and then use them as targets for DVS data to do supervised learning with tiny YOLO architecture. The result shows that purely using DVS data, object detection can reach a truly high speed (100 FPS) in a real environment. Although the processing speed is satisfactory, the accuracy of the detection is equally essential. For multi-sensor based object detection, different modalities are often combined to achieve higher detection accuracy (Enzweiler and Gavrila, 2011; Gupta et al., 2014; Premebida et al., 2014; Chen et al., 2016; Schlosser et al., 2016). There have been many kinds of fusion methods used in object detection. For instance,we can simply map different inputs together, use probabilistic fusion or concatenation fusion. González et al. (2017) fuse RGB and depth images using both early fusion and late fusion methods and their multimodal framework obtains significant accuracy improvement. However, non-overlapping regions and uncertainties can put early fusion into trouble. Chavez-Garcia and Aycard (2016) proposed a late fusion method based on the Evidential framework which can enhance the description of the objects to reduce uncertainties.

In this paper, we are trying to unlock the potential of event data on object detection. However, the output of event-based sensors is an event stream instead of the sequence of frames, which is entirely different from frame-based sensors. Since we cannot use standard computer vision algorithms to process such data directly, new methods need to be proposed to cope with the temporal contrast instead of absolute brightness representing the value of each pixel. Considering the low frame rate of APS would lower the detection speed (Liu et al., 2016a), we decide to purely use event-based data for object detection by introducing three event-stream encoding methods. Additionally, since fusion methods can make detectors more robust (González et al., 2017), we proposed channel-level fusion and decision-level fusion with multi-cue event information (Dollár et al., 2009). In the end, we developed two pedestrian detectors. To evaluate the performance, we perform several experiments with different combinations of sensor data and algorithms. Conducive discussions based on our experiments are presented. Hopefully, our work can inspire relevant research and pave the way of more fascinating perception applications with neuromorphic vision sensors.

The remainder of this paper is organized into three parts. Section 2 presents the methodology of our pedestrian detection system with multi-cue event information fusion. Section 3 presents the experiments carried out to assess our proposal step by step, and discuss the obtained results. Finally, section 4 draws our conclusion.

## 2. Methodology

**Figure 1:**
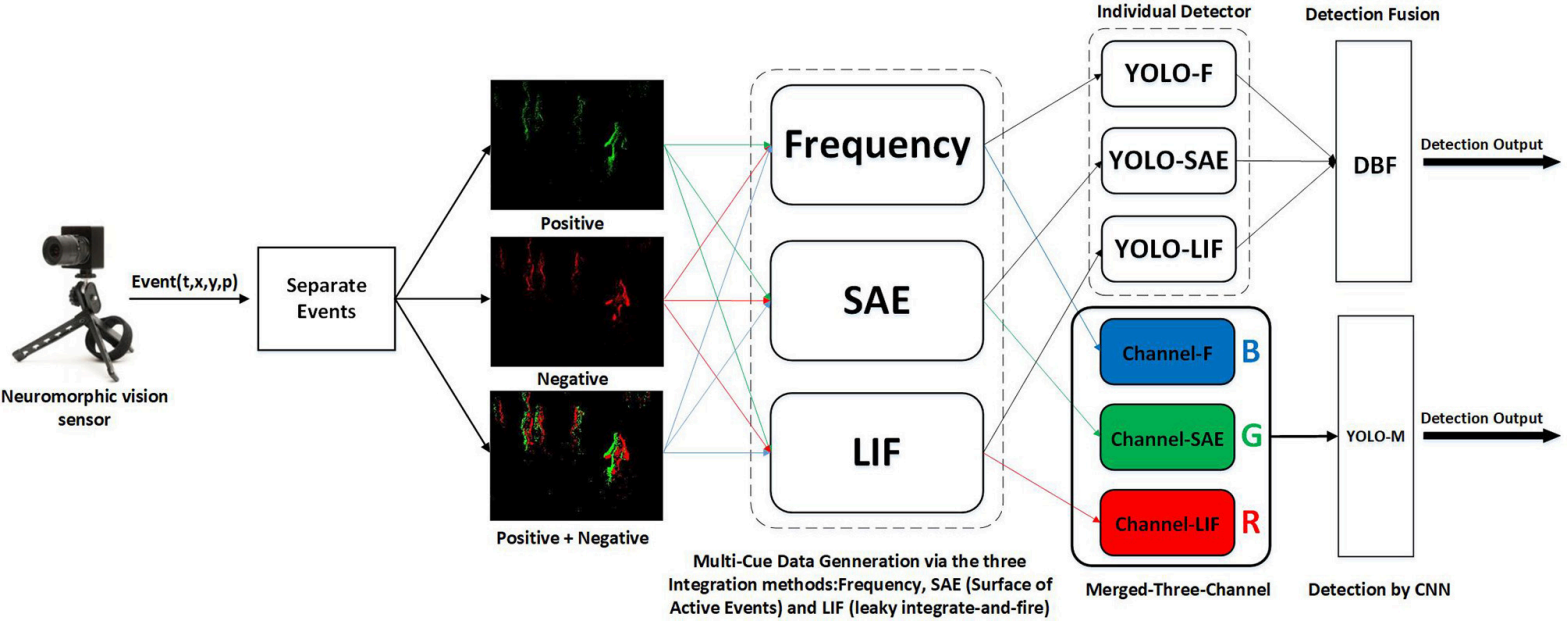
Overview of the pedestrian detection system with multi-cue event information fusion. *Frequency, SAE (Surface of Active Events) and LIF (leaky integrate-and-fire)* are the three methods we use to encode event stream. And we encode Positive, Negative, and Positive + Negative events respectively. We do channel-level fusion by corresponding these three encoding methods to R, G, B channels respectively, then by processing the merged data we obtain a detector. Moreover, we process different encoded data separately to acquire three detectors, then we use DBF to do decision-level fusion and obtain one fused detector.

Our approach aims at detecting pedestrians using neuromorphic vision sensor. As shown in Figure 1, we introduced three encoding methods which convert event stream to event frames over a constant time interval. Then, a standard deep neural network with input from the event frames is utilized to predict the locations of pedestrians. Different fusion strategies are further investigated to improve the performance of our detection system. We detail the methodology of our system and implementation as follows.

### 2.1. System Overview

The architecture of the proposed pedestrian detection system with multi-cue event information fusion is shown in Figure 1. We first split neuromorphic vision sensor data into three parts based on the event polarities: positive event streams, negative event streams, and all event streams (fusing positive and negative event streams). The purpose is to figure out the influence of the event polarity on detection performance. In order to take full advantage of the event information, we propose the idea of multi-cue event information fusion. To be specific, three Event-to-Frame encoding methods, *Frequency, SAE (Surface of Active Events) and LIF (leaky integrate-and-fire)* are applied to event streams respectively and generate three sequences of event frames. These encoding methods are chosen to reflect different characteristics of the event stream.

The three event frame sequences would be synthesized with two fusion approaches: the channel-level fusion and the decision-level fusion. The former approach aims to synthesize the data by using the three sequences as the RGB channels of an image. The RGB images are then used as the input of the renowned neural network architecture YOLO (Redmon et al., 2016) to acquire a pedestrian detector. The latter approach, however, respectively feeds the three sequences of event frames to YOLO to obtain three individual detectors. These detectors are then fused by a DBF (Dynamic Belief Fusion) function (Lee et al., 2016) to achieve better detection performance.

### 2.2. Multi-Cue Information Generation

**Figure 2:**
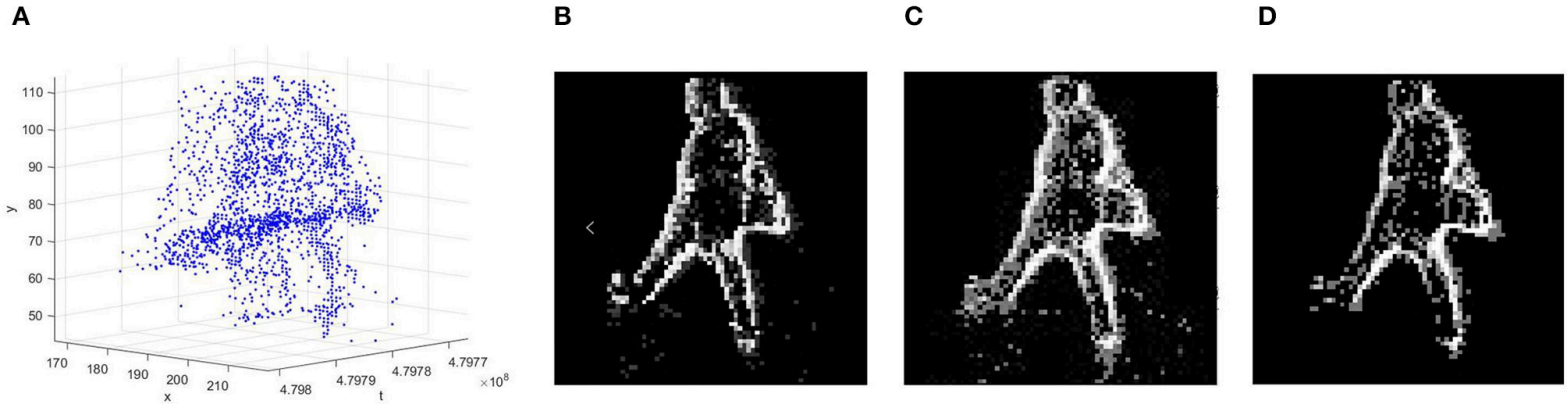
**(A)** The representation of the spatiotemporal data from neuromorphic Vision Sensor in 3D(x, y, t), **(B)** event-frame based on Frequency **(C)** event-frame based on SAE, **(D)** event-frame based on LIF.

In our work, an event-to-frame conversion is carried out to encode the event stream for pedestrian detection, and we deployed the frames with a state-of-the-art deep learning algorithm. We employ three different event-stream encoding methods, which are selected according to their ability to reflect different aspects of the event information. By binning the neuromorphic sensor's outputs in 20 ms interval, the continuous event streams are converted to a sequence of image frames, and the pixel values of each image are encoded via the three different Event-to-Frame encoding methods: *Frequency, SAE (Surface of Active Events) and LIF (leaky integrate-and-fire)*. Figure 2 shows the encoded frames at the same moment with different event-stream encoding methods. Although encoding approaches are employed as extra work to convert event streams to event frames, it is still possible to achieve high-speed detection by using a sliding window with a specific time interval. The following part illustrates the details of the employed approaches.

#### 2.2.1. Event-Stream Encoding Based on Frequency

Considering that the occurrence frequency of an event within a given time interval can represent its probability to be a valid event instead of noise, we count the event occurrence at each pixel (*x, y*), based on which we calculate the pixel value using the following range normalization equation inspired by (Chen, 2018):

(1) $$\begin{array}{r} \sigma \left(n\right)=255\cdot 2\cdot \left(\frac{1}{1+e^{-n}}-0.5\right) \end{array}$$

where *n* is the total number of the occurred events (*positive or negative*) at the same pixel coordinate (*x, y*) within 20 ms interval, and σ(*n*) is the pixel value of the event frame. It can be noticed that we normalize the range of σ(*n*) to between 0 and 255 in order to fit the 8-bit image.

What inspires us to apply this encoding method is that the edges of a moving object tend to be the edges of the illumination in the image. Thus there would be much more event occurred near the object's edges.

Therefore, if we utilize the event frequency as the pixel value, the edges of the object would be strengthened to a great extent, which is beneficial for object detection as we have a more clear profile of the object. Moreover, as sensors usually have noise, one of the superiority of this encoding method is to filter out the noise. As is shown in Figure 2B, the outline of the pedestrian is highlighted while the noise point is significantly reduced.

#### 2.2.2. Event-Stream Encoding Based on SAE (Surface of Active Events)

Due to the extremely low latency, the neuromorphic vision sensor can record the exact occurred time of every incoming event, which is a unique advantage of neuromorphic vision sensors over traditional ones. In order to take full advantage of such characteristic, we apply the event-to-frame approach called SAE (Surface of Active Events) (Mueggler et al., 2017), where the pixel values of a frame are determined directly by the occurrence time of the events. Specifically, each incoming event [*t, x, y, p*] will change the pixel value *t*_*p*_ at (*x, y*) according to the time-stamp *t*, and thus an image frame given by the time-stamp of the most recent event at each pixel is acquired:

(2) $$\begin{array}{r} SAE:t\Rightarrow t_{p}\left(x,y\right) \end{array}$$

Moreover, to attain an 8-bit single channel image, numerical mapping is conducted by calculating the Δ*t* between the pixel value *t*_*p*_ and the initial time *t*_0_ for each frame interval *T* as follows:

(3) $$\begin{array}{r} g\left(x,y\right)=255\cdot \frac{t_{p}-t_{0}}{T} \end{array}$$

The reason why we employ this encoding approach lies in its superiority in reflecting time information because the raw timestamp information is directly utilized while the pixel value and its gradient can tell the moving direction and speed of the event stream. The acquired gray image of a scene with pedestrians is shown in Figure 2C. It is apparent that the motions of the pedestrians are recorded, and the gray value of each pixel indicates the occurrence time during the frame interval. The main shortcoming of such approach is its inability to filter out noise due to the ignorance of frequency information. Also, this method has requirements for movement speed considering that a newly arrived event would cover up previous pixel values.

#### 2.2.3. Event-Stream Encoding Based on LIF Neuron Model

**Figure 3:**
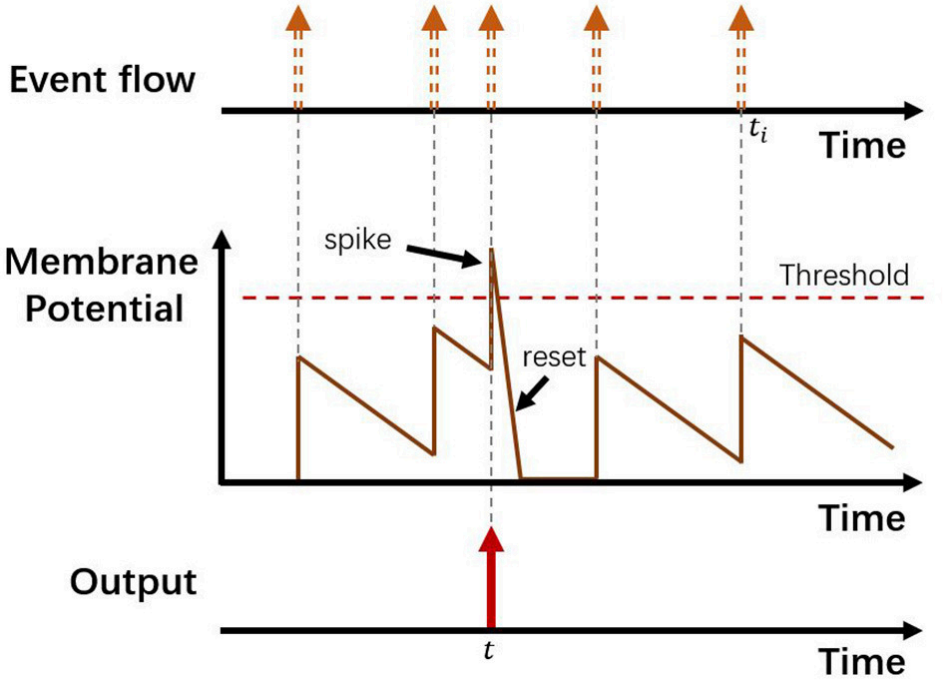
The encoding procedure of the LIF neuron model. Top shows an asynchronous event stream. At time *t*, there is a spike of the LIF neuron.

The third encoding method we leveraged is based on the LIF (leaky integrate-and-fire) neuron model (Burkitt, 2006). According to the LIF model, each neuron has its own Membrane Potential (MP) which will be influenced either by input spikes or time-lapse, and a firing spike output would be generated if the MP exceeds the preset threshold. In our case, every image pixel (*x, y*) is regarded as a neuron with its Membrane Potential and firing counter *n*. Each incoming event at (*x, y*) will cause a step increase of the pixel's MP regardless of its polarity, and simultaneously each pixel's MP will decay at a fixed rate. Also, we selected a proper threshold for MPs. In a specific time interval, we count the number of times each pixel's MP exceeds the threshold (recorded as *n*), and once a pixel's MP exceeds the threshold, the MP will be reset to 0 with no latency. Then we do range normalization by using Equation 1 to acquire the corresponding pixel value. After each interval, the firing spikes counter *n* of each pixel will be reset to 0. The encoding procedure of the model is illustrated in Figure 3.

Our motivation for applying this encoding is derived from its time-continuous characteristic and the ability to reflect occurrence intensity of events. With the time-continuous nature of the pixel MP, historical event data have been taken into account so that the output frames contain more abundant information without the limitation of the time interval. Moreover, the event intensity can be indicated because firing spikes are generated only with input intensive enough to break through the threshold. In this case, the noise will also be filtered to a great extent.

### 2.3. Network Architecture

YOLO, the acronym of “You Only Look Once,” is an object detection system which was first put forth by Redmon et al. (2016). Its latest version YOLOv3 by Redmon and Farhadi (2018) has state-of-the-art performance tested on COCO test-dev. Comparing with other detectors, YOLOv3 performs the best with the measurement of IOU@0.5 mAP. In YOLO object detection system, an individual neural network is applied to the whole image at the same time. By such a neural network, the image is divided into grid cells called “regions,” and the prediction of bounding boxes and probabilities for each region of the image is made. YOLO looks at the whole image at the same time, so its predictions gain information concurrently by the global context in the image.

YOLO object detection system has a model size modification mechanism, with which one could find trade-off point between speed and accuracy. More specifically, modern YOLOv3 has three primary model size: 320, 416, and 608. The tiny model has the minimum requirements of computation, and with the most rapid detection speed that makes this model remarkably applicable for embedded systems and real-time detection jobs, while the largest model needs more computational resources while performs the best on mAP.

In this work, we choose the standard YOLOv3-416 model as our base model. Specifically, the input image is resized to the resolution of 416 × 416 pixels. Then, a single convolutional network runs on the image to predict object bounding boxes. The network takes DarkNet-53 which comprises 53 convolutional layers as the backbone network and adds residual layers as shortcut connections. After that, fully connected layers are used to connect a set of bounding box outputs. Finally, a non-maximum suppression is applied to suppress duplicated detection. YOLOv3 looks at the whole image to train detection model which can realize real-time prediction of bounding boxes and their class probabilities simultaneously.

To be concise, we adopt a unified naming method to shorten the length of the names of our YOLO-based neural networks. The event frames converted by three encoding methods, namely Frequency, SAE and LIF, are used as individual inputs to the standard YOLOv3-416 model. Hence the trained networks are referred as YOLO-F, YOLO-SAE, and YOLO-LIF respectively.

### 2.4. Multi-Cue Fusion

#### 2.4.1. Channel-Level Fusion

**Figure 4:**
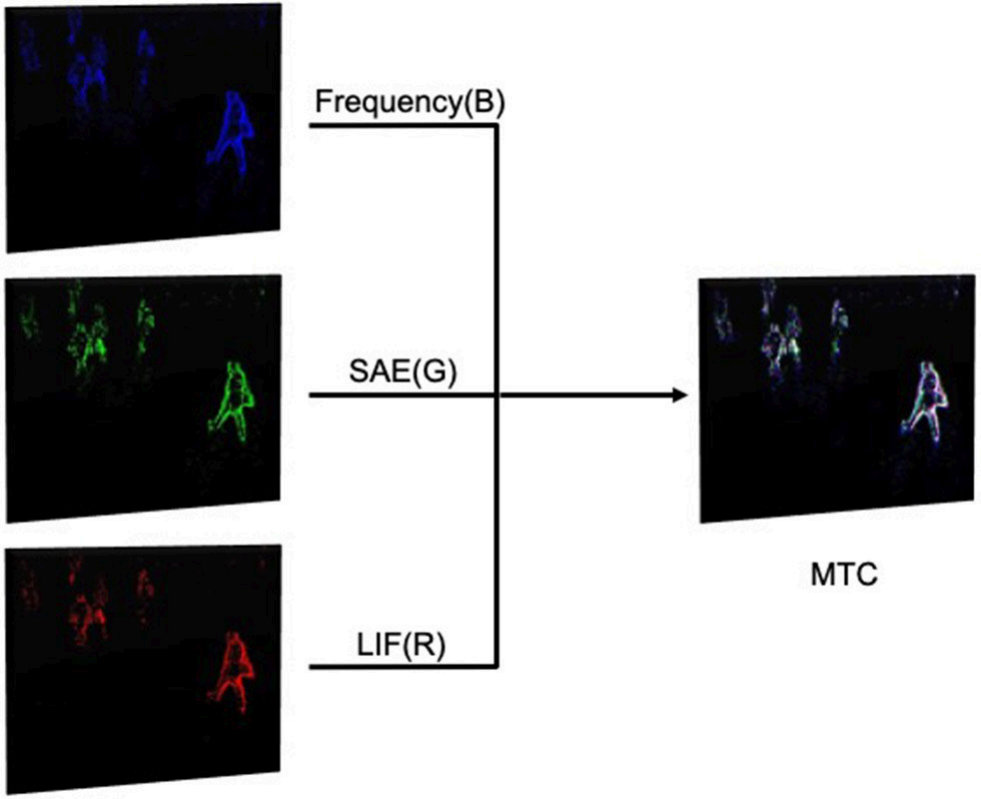
The model of the Merged-Three-Channel method: we colorized three before-merging event frames for better effect of visualization, the actual frames used in this work are grayscale.

We deployed a method called *MTC (Merged-Three-Channel)* to achieve Channel-Level Fusion. Specifically, we merged three corresponding event frames using the three presented encoding mechanisms, namely *Frequency, SAE, and LIF*. The definition of the three channels in the merged frame is [B, G, R] = [Frequency, SAE, LIF], as shown in Figure 4. The merged frame is of the OpenCV Matrix type of 8UC3, which means that the frame is an 8-bit unsigned integer image with three channels.

The advantages of each encoding method are illustrated in section 2.2. It is noted that each encoding method has a different focus of input data so that they are able to complement each other and be adaptive to different application scenarios. Here we synthesize the three channels together aiming to make the most of the event stream information. It is expected that the pedestrian detector based on such merged data could enjoy better adaptability and robustness with a higher average detection precision. For example, the SAE is likely to perform the best with high speed moving objects while the other two approaches perform better with serious noise impact.

To implement channel-level fusion, the event frames converted by MTC methods are also implemented as individual input to the standard YOLOv3-416 model. Thus the trained networks are referred as YOLO-MTC.

#### 2.4.2. Decision-Level Fusion

Dynamic Belief Fusion (DBF) in Lee et al. (2016) is a state-of-the-art algorithm for the fusion of heterogeneous object detection methods. In this work, we use DBF to fuse detection results from three individual YOLO-based detectors, YOLO-F, YOLO-SAE, and YOLO-LIF. DBF relies on Dempster-Shafer Theory (DST) which consists of Shafer's belief theory and Dempster's Combination Rule. Shafer's belief theory gets a degree of belief for a hypothesis, and then DST combines such beliefs from multiple independent sources via Dempster's Combination Rule. DBF fuses the output of multiple approaches to improve accuracy.

In binary object detection, we can get a set *X* which is composed of {∅, *T*, ¬*T*, {*T*, ¬*T*}}, where *T* is a target hypothesis, {*T*, ¬*T*} represents intermediate state and ¬*T* is a non-target hypothesis. According to the detection outputs of three individual detectors, YOLO-F, YOLO-SAE and YOLO-LIF, we can draw Precision-Recall curve. And a theoretical best possible detector proposed in Lee et al. (2016), ${\hat{p}}_{bpd}$, whose *P* − *R* curve is modeled as

(4) $$\begin{array}{r} {\hat{p}}_{bpd}=1-r^{n} \end{array}$$

where *r* is recall. Then, we can get the corresponding recall *r* and the corresponding precision (*p*) by mapping detection scores of different detectors. *p* is assigned as the basic probability of target hypothesis(*m*(*T*)), *m*(*I*), the intermediate state hypothesis, is defined by ${\hat{p}}_{bpd}-p$ and the precision $1-{\hat{p}}_{bpd}$ is assigned to *m*(¬*T*) which represents non-target. After that, DBF use Dempster's combination rule to compute fused basic probability of target and non-target hypotheses *m*_*f*_(*T*) and *m*_*f*_(¬*T*). Lastly, a fused confidence score *s* is returned by Shafer's belief theory. See Lee et al. (2016) for more details.

The purpose of applying DBF is to conduct a comparison with the presented MTC fusion method and to explore the effects of the decision-level fusion algorithm framework and channel-level fusion algorithm framework on our data. Our goal is to take advantage of the potential information of event streams derived from neuromorphic vision sensors to achieve higher detection accuracy. Since DBF can robustly extract complementary information from multiple detectors and generate better performance over the best individual detector in traditional object detection mission, we use DBF to fuse the detection outputs of multiple detectors which are based on three event-stream encoding methods. Our wish is to inspire greater efforts along the lines of fusion research on neuromorphic vision sensor.

## 3. Experiments

The experiments of pedestrian detection with our proposed approaches were conducted on a self-annotated dataset. The following section presents detailed information about the implementations of our proposed detection system as well as its performances. Qualitative and quantitative analyses and discussions of the experiment results are provided.

### 3.1. Dataset

Based on our knowledge, there is no public labeled pedestrian dataset created with a neuromorphic vision sensor. Therefore, we decided to create a dataset by ourselves. The dataset was collected by a neuromorphic camera named DAVIS240 which was mounted on the second floor of Munich main railway station. The DAVIS240 was titled toward the pedestrians walking on the first floor. The dataset includes four raw event streams which are recorded from different views and locations. The length of the dataset is 485.2 s containing 1249.5 M events. The resolution of the camera is 240 × 180. It is worth to note that we also manually split the event stream to two parts during our experiments. For each raw event stream, 2,500 short event streams with a time interval of 20 ms are created. In total, 8736 short event streams are annotated by ourselves. In which, there are 28,109 bounding boxes labeled as pedestrians and there are around 3.22 pedestrians on average in each frame. Nomenclature of the dataset groups are listed in Table 1.

**Table 1:** Nomenclature of the groups.

**Notation**	**Definition**
P	All events contained in the frame is positive.
N	All events contained in the frame is negative.
PN	All events contained in the frame is positive or negative.
Frequency	Encoded by the method of Frequency.
SAE	Encoded by the method of Surface of Active Events.
LIF	Encoded by the method of Leaky Integrate-and-Fire.
MTC	Encoded by the method of Merged-Three-Channels.

### 3.2. Implementation

#### 3.2.1. Channel-Level Fusion

On configuring neural networks of YOLO, we modified the .cfg configuration file which defines the neural network, the data augmentation, and training rules.

We select the standard YOLOv3-416 network, configure the network to be compatible with a single class, set the batch and subdivision to be 1 with max batches to be 50,000, and propose default data augmentation method. The initial learning rate is set as 0.0001; if the learning rate is too high (as default, 0.001) for this dataset, the neural network will never converge during training. On training YOLO, we select 6,989 annotated frames (~80%) as the training set, and 1,747 annotated frames (~20%) as the validation set. All frames are aligned to the size of 416px × 416px initially before training to accommodate the network input. We implemented the YOLO training with Nvidia^TM^ Titan X GPUs and saved weights of each 10,000 training iteration for further evaluation processes. Same training configures are implemented on all 12 groups in the EPedestrian dataset used for training.

#### 3.2.2. Decision-Level Fusion

In this work, our detecting object is pedestrian which is a binary object detection task. We use the YOLO-based CNN framework to get the detection results from three different event-stream encoding methods Frequency, SAE, and LIF. Then, 2D object bounding boxes and detection scores from three YOLO-based object detectors (YOLO-F, YOLO-SAE, and YOLO-LIF) are used as inputs for DBF mentioned in section 2.4.2. The *P*−*R* cure is calculated by ground truth and detection results. Moreover, we choose *n* as 18 to model the perfect detector in Equation (4). Although according to Lee et al. (2016), the parameter *n* should be set to infinite for the notional perfect detector, we discover that the performance of the fused detectors would not have noticeable improvement with *n* > 18 but suffer dramatically increasing time consumption for calculation. So we set *n* = 18 for a balance between runtime and accuracy.

### 3.3. Performance Evaluation

**Figure 5:**
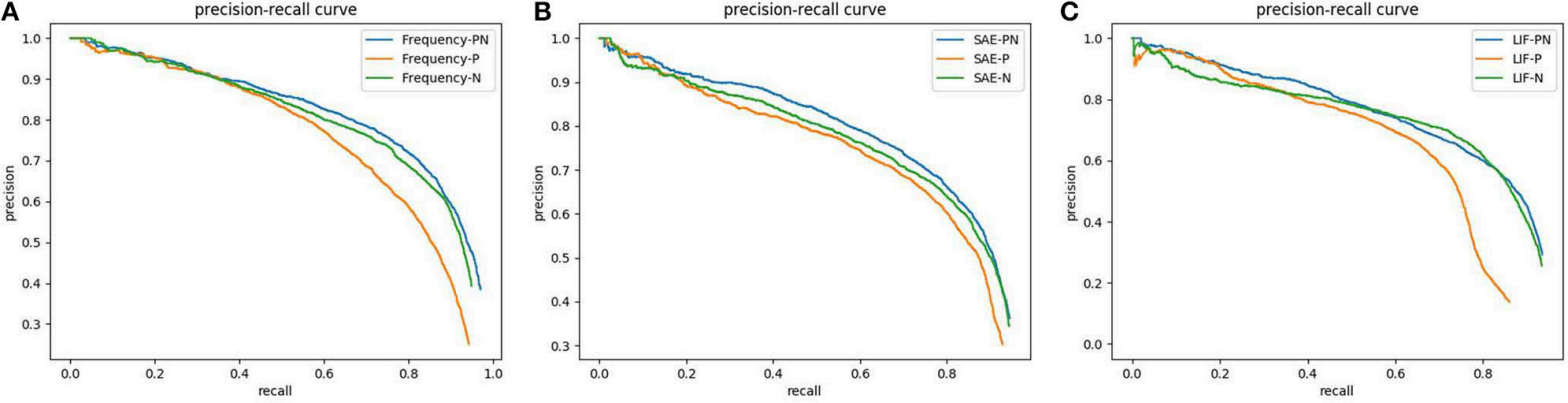
Precision-Recall curves of different event-stream encoding methods (Frequency, SAE and LIF) based on YOLO with different Positive-Negative combination dataset: **(A)**YOLO-F; **(B)** YOLO-SAE; **(C)** YOLO-LIF.

**Figure 6:**
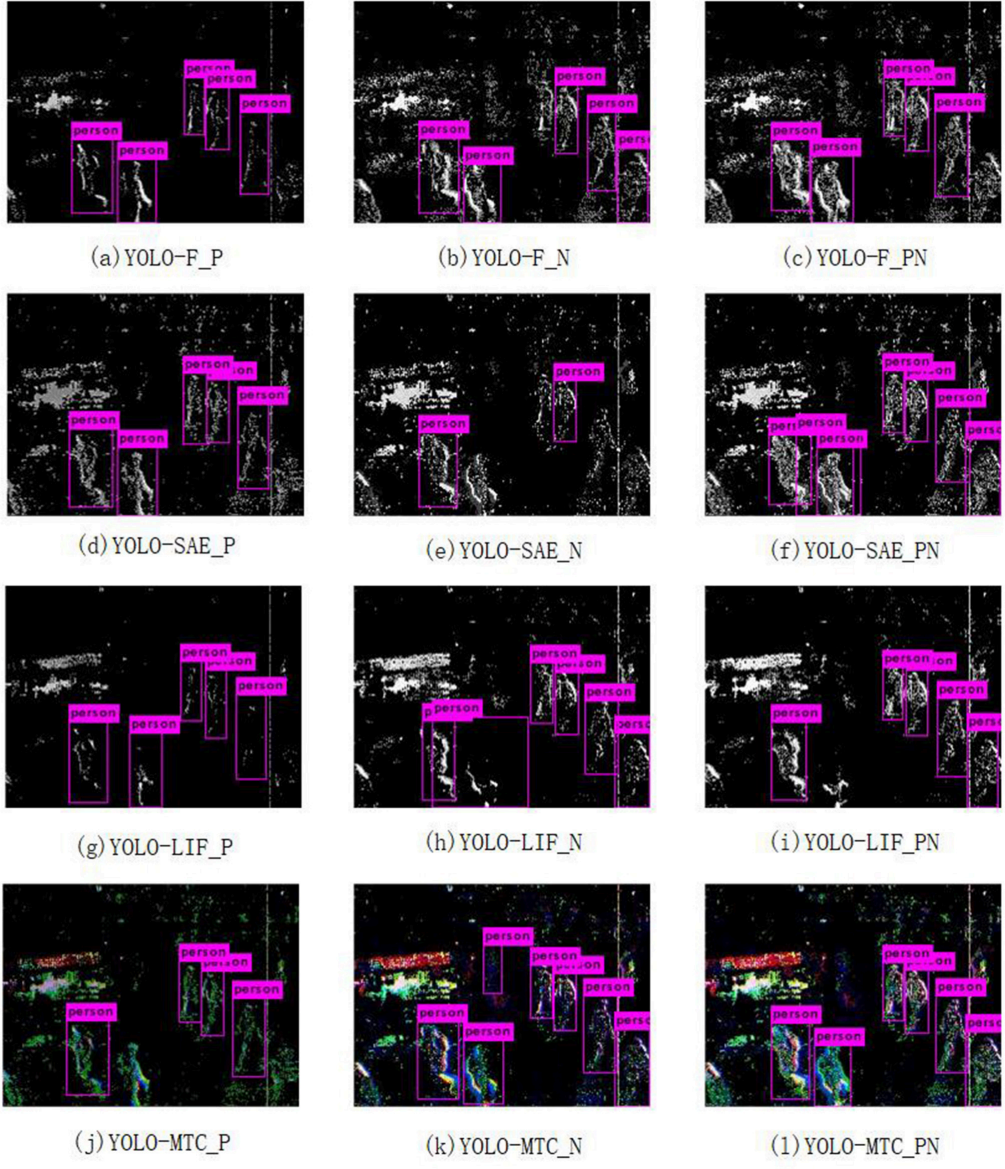
Predicted results: The outputs from three individual detectors (YOLO-F, YOLO-SAE and YOLO-LIF) are shown in the upper three rows while outputs from YOLO-MTC are shown in the lower row. Meanwhile, the results of the same Positive-Negative combination dataset are shown in the same column. **(A)** YOLO-F_P; **(B)** YOLO-F_N; **(C)** YOLO-F_PN; **(D)** YOLO-SAE_P; **(E)** YOLO-SAE_N; **(F)** YOLO-SAE_PN; **(G)** YOLO-LIF_P; **(H)** YOLO-LIF_N; **(I)** YOLO-LIF_PN; **(J)** YOLO-MTC_P; **(K)** YOLO-MTC_N; **(L)** YOLO-MTC-PN.

YOLOv3 is trained with 12 different groups of event frames separately. The average precision (AP) of the models evaluated on EPedestrian Dataset are provided in Tables 2, 3. The Precision-Recall curves of three individual detectors, YOLO-F, YOLO-SAE, and YOLO-LIF, are shown in Figures 5A–C respectively. The pedestrian detecting results which are separately obtained by YOLOv3 in a train station hall with a bakery scene are presented in Figure 6. We illustrate our experimental results in the following three aspects and the detailed discussions and analyses will be presented in the next section.

**Table 2:** The performance for polarity datatset using different event-stream encoding methods (Frequency, SAE, and LIF) based on YOLO (YOLOv3 IOU@0.5 AP).

**Detector**	**Polarity**	**AP**
YOLO-F	Positive	**74.48%**
YOLO-SAE	Positive	71.76%
YOLO-LIF	Positive	63.05%
YOLO-F	Negative	**78.62%**
YOLO-SAE	Negative	74.25%
YOLO-LIF	Negative	71.05%
YOLO-F	Positive + Negative	**81.04%**
YOLO-SAE	Positive + Negative	76.47%
YOLO-LIF	Positive + Negative	72.72%

*The bold values significant the best value of encoding methods in same polarity group*.

**Table 3:** The performance for Positive-Negative combination dataset using different event-stream encoding methods (Frequency, SAE and LIF) based on YOLO and two fusion strategies.

**Detector**	**AP-P**	**AP-N**	**AP-PN**
YOLO-F	74.48%	78.62%	81.04%
YOLO-SAE	71.76%	74.25%	76.47%
YOLO-LIF	63.05%	71.05%	72.72%
YOLO-MTC	76.06%	77.26%	78.98%
DBF	78.53%	80.86%	82.28%

#### 3.3.1. Comparison on Multi-Cue Information

We studied the three event-to-frame encoding methods with the data stream of different polarity. Average precision (AP) of the models with different encoding methods and event polarity is illustrated in Table 2. It is noted that the detector based on Frequency encoding, i.e., YOLO-F accomplishes the best performance among single-channel detectors regardless of event polarity. Following YOLO-F as the best, YOLO-SAE outperforms YOLO-LIF with data of all polarity. Moreover, evident gaps of performance exist among the three single-channel detectors in the order of YOLO-F, YOLO-SAE, and YOLO-LIF regardless of event polarity. Among these results, the YOLO-F with positive-negative events achieves the highest AP of **81.01%**, which is a quite high accuracy even if comparing with state-of-the-art pedestrian detectors. As is shown in Figure 6, the first three rows exhibit the detection results of the YOLO-F, YOLO-SAE, YOLO-LIF. If we ignore the influence of event polarity and focus on the third column, we can notice that the missing or wrong bounding boxes tend to be different within the three rows, which can be attributed to the complementary nature of the three encoding methods.

#### 3.3.2. Effect on Positive-Negative Combination

The effect of event polarity on detection performance can be reflected in the Precision-Recall curves shown in Figure 5. As PR curves, the value of AP equals to the area of the closed region surrounded by the x-axis, the y-axis, and the precision-recall curve. In short, the larger the closed area or higher the PR curve is, the better performance of CNN-based pedestrian detection achieves. It is evident in Figure 5 that the positive event stream performs the worst regardless of the employed encoding methods. Meanwhile, the positive-negative combined data achieve a better performance than the negative one but the discrepancy is quite small. As is shown in Figure 6, each column presents different polarity of the input data in the order of positive, negative and combined one. Regardless of the encoding methods, it is noticeable that the amount of events in the positive column is evidently less than the other two, while the difference between negative and combined one is quite inconspicuous.

#### 3.3.3. Performance Evaluation on Different Fusion Strategy

In Table 3, the detection results of the three single-channel detectors and the two fusion methods are presented. Regardless of the polarity of the data, DBF outperforms both MTC fusion and the three single-channel detectors on our dataset, and the best AP value of it reaches **82.28%**, better than the 81.04% of YOLO-F. The performance improvement of DBF indicates that detection accuracy can be improved by investigating complementary information provided by each detector. As for the other fusion approach, MTC fusion performs better than all the three single-channel detectors only with positive event data. It is surprised that the average accuracy of MTC fusion is lower than YOLO-F in both negative and positive-negative event data. This result apparently fails to meet our expectations. In Figure 6, the detection results of the MTC fusion is illustrated in the last row.

### 3.4. Discussion

As an attempt to make full use of the event information of the neuromorphic vision sensor, we not only propose the multi-cue fusion concept and conduct experiments based on it, but also provide relevant analyses and explanations of the results, hoping to clarify the idea of multi-cue fusion further and inspire relevant work.

#### 3.4.1. What Can We Learn From the Evaluation of Different Event Polarity?

The Precision-Recall curves shown in Figure 5 tells us that the polarity-combined event frame achieves the best performance while the positive one the worst for all the encoding channels. We explain the excellence of the combined frame by the fact that the polarity-combined event frame collects more information than single polarity one, and such abundance of information is beneficial to the network training. In contrast with the positive channel, the negative one realized a much closer PR curve to the combined channel. This result can be explained by the difference of event amount between the two polarities. We assume that the further reason behind it might lie in the characteristics of human walking posture: the longer contour of the back than of the front. This inspires us to give preference to the negative events for pedestrian detection when we want to reduce the input data but achieve similar performance.

#### 3.4.2. Why Does YOLO-F Perform Better Than the Other Two Single-Channel Detectors?

It is noted in Table 2 that the detector based on the frequency channel performs better than the other single-channel detectors. We explain this phenomenon by the hypothesis that the frequency-based method is a more general and all-around encoding method which is quite appropriate for the constructed pedestrian dataset. For comparison, the SAE-based encoding method is limited to time information so that it fails to filter out any noise event, while the LIF-based method set a stricter rule for event recording, leading to less noise but correspondingly increasing loss of valid information. As for the frequency-based one, it makes a balance between noise and valid information. Simultaneously, the unique time-sensitive and intensity-sensitive advantages of SAE-based and LIF-based exert limited influence on the detection performance here because the applied dataset reflects little information of speed or intensity.

#### 3.4.3. Why Does the Channel-Level Fusion Not Perform as Expected?

As shown in Table 3, the merged-channel detector, i.e., YOLO-MTC does not live up to our expectation and only achieve a mediocre performance comparing to YOLO-F and DBF. The reason for the unsatisfactory performance of the Channel-Level fusion is that all event frames are seen as a three-channel RGB image, while gray-scale images duplicate its single channel to three. Such procedure makes the method behaves more like averaging the advantages of the information of its three channels so that networks based on Merged groups will perform averagely, taking into account the performance gaps among the three single-channel detectors.

However, the unsatisfactory results can be attributed to the lack of dataset with a complex environment as well. Considering that the significant advantage of the Channel-Level fusion lies in its adaptation and robustness, it is not fair to judge its performance with relatively similar scenarios. We still believe that this method is worth a try in some situation, especially when the features of the recorded events are constantly changing such as with erratic weather, variable object moving speed or changing brightness. The chances are that the three channels could complement and reinforce each other to achieve the best performance. Therefore, it is an inspiration to employ the Channel-Level fusion to a more volatile environment.

#### 3.4.4. What Makes Decision-Level Fusion Outperform the Other Methods?

The average precision (AP) in Table 3 shows that DBF outperformed MTC as well as individual detectors on our pedestrian dataset. We guess that CNN performed much worse than DBF because coarse-grid scanning windows and aspect ratio of windows fixed as square bring localization error. By integrating multi-cue information, DBF can reduce the inaccurately localized problem and improve the detection accuracy.

As is analyzed in Lee et al. (2016), DBF can guarantee an improved performance of detection over the best detectors in the fusion pool, because it would always return the bounding box with the highest detection score among all the detectors' results. However, the system runtime would keep increasing with additional detectors because input data need be fed to all detectors respectively before decision fusion. Therefore, in order to seek a balance between accuracy and efficiency, the choices of additional detectors are significant. Only with detectors whose advantages are complementary, could the fused one achieve better detection performance with relatively low time consumption. That is also the purpose of our selection of the three encoding methods, which all contribute to the excellent performance of the Decision-Level Fusion.

#### 3.4.5. What Is the Inspiration of Our Work for Future Research?

Although the focus of our work is laid on pedestrian detection, it is conceivable that the proposed principle of multi-cue event information fusion is applicable for many other perception tasks of neuromorphic vision sensors. For instance, detection of different objects besides pedestrian, feature extraction, and tracking, multi-object tracking, etc. It is also worth noting that given an extra frame encoding procedure, high-speed detection is still achievable by using a sliding window with a certain time interval. Hence the low-latency superiority of neuromorphic vision sensors can still be retained. The excellent performance of the detector based on the decision-level fusion inspires that we can improve fusion performance by increasing detectors with higher detection accuracy and decreasing detectors with lower detection accuracy. Simultaneously emphasis should be paid to the complementary relation among the selected detectors.

In this paper, we deploy three different event-to-frame encoding methods to unleash the potential of event data on application of neuromorphic vision sensor. Thinking further, we can develop algorithms that directly process the event data generated from neuromorphic vision sensor as well. Processing the event data directly may improve running speed and reduce the computational cost. Additionally, we make the summation time as a constant in this work (here 20 ms), while different time intervals can also be tried in further research to fulfill different demands. In our future work, we plan to construct an event-based dataset with more abundant detection objects and a more complex environment, so that we can test the adaptation and performance of our presented detection system in different situations, especially the channel-level fusion. Besides, more appropriate event-stream encoding methods and the integration of accurate detection algorithms without compromising computational speed are the main tasks of our future work.

## 4. Conclusions

With the purpose of improving pedestrian detection accuracy with neuromorphic vision sensors, we put forward the idea of multi-cue event information fusion. Based on such a principle, we introduced several encoding methods with different advantages of reflecting event information, after which two possible data fusion approaches are presented. In addition, two pedestrian detection systems based on multi-cue fusion are developed. It is noted that all of these approaches share the same motivation: to unlock the potential of event stream data in different situations. The thorough evaluation of the proposed detection system is then carried out, and we also provide detailed analyses of the results and prospect of future work. Hopefully, our work could contribute to the perception applications with neuromorphic vision sensors.

## Data Availability

The datasets generated for this study are available on request to the corresponding author.

## Author Contributions

GC, JC, FR, XM, and AK did the conception and design of the manuscript. GC, HC, CY, ZZ, XL, and ZQ did the analysis and interpretation of data, drafting and revising the article. JC provided the sensor. GC and ZQ acquired the data.

### Conflict of Interest Statement

The authors declare that the research was conducted in the absence of any commercial or financial relationships that could be construed as a potential conflict of interest.
